# The climate change dilemma of ski destinations: adaptation becomes maladaptation

**DOI:** 10.3389/fspor.2026.1865169

**Published:** 2026-08-18

**Authors:** Ching Li, Ting-Yen (Tim) Huang, Jia-Rui Zheng

**Affiliations:** 1 Graduate Institute of Sport, Leisure, and Hospitality Management, National Taiwan Normal University, Taipei City, Taiwan; 2 Department of Leisure Industry and Health Promotion, National Ilan University, Yilan City, Taiwan

**Keywords:** adaptation, climate change, Japan, maladaptation, ski destination, ski tourism, snow reliability, sustainable sport policy

## Abstract

Climate change is progressively eroding the operational viability of ski tourism, while measures intended to sustain winter seasons can generate new vulnerabilities. This study examines how technical interventions, infrastructure reinforcement, and activity-expansion strategies shape adaptation and maladaptation risk in Japan's ski sector. We develop an exploratory Snow Reliability Line (SRL) screening framework that links elevation-based snow-reliability exposure, publicly documented resort actions, and maladaptation coding. The exposure framework is calibrated with Japan Meteorological Agency (JMA) 1991–2020 station climatology and tested through multi-parameter sensitivity analysis. It combines Alpine SRL research with a Japan-oriented regional adjustment and a document-based assessment of adaptation and maladaptation pathways. The maladaptation evaluation relies exclusively on conservative, document-based policy analysis of publicly available resort materials. The exploratory classification reveals a spatial and operational divide. High-elevation and northern resorts retain relatively stable winter conditions, whereas low- to mid-elevation sites are more frequently classified as snowmaking-dependent or critically marginal. Maladaptation risks are concentrated in the A1-B1-Dead and A1-B1-C1 pathways, where activity expansion, extended operations, accessibility incentives, and fixed winter infrastructure are associated with higher aggregate energy demand, opportunity costs, and path dependence. The findings show why ski-destination adaptation should be evaluated together with mitigation, anticipatory governance, investment flexibility, and year-round carbon accounting. Among the 14 resorts in the two highest-risk pathways, six show no documented transition planning, indicating potential continuity-driven maladaptive exposure distinct from action-driven lock-in.

## Introduction

In recent years, warm winters have become more frequent, and the spatiotemporal stability of snowfall has weakened markedly, reshaping the distribution of snow resources and placing increasing pressure on snow-dependent ski tourism. In Japan, the combined effects of a pronounced north–south climatic gradient, complex topography, and strong regional variation in ski resort development make low- to mid-elevation areas particularly sensitive in terms of operating windows, snow conditions, and cost structures (1–5). At the same time, Japanese ski destinations range from small locally embedded resorts to large corporate operators, and their capacities to absorb climatic pressure, invest in snowmaking, or diversify into four-season business models are far from uniform (6). Under these conditions, evaluating adaptation measures solely by whether they can “save the season” is no longer sufficient.

Absent accompanying changes in energy systems, transport systems, and destination governance, technical responses that appear effective in the short term—such as artificial snowmaking, season extension, or infrastructure reinforcement—may generate new forms of vulnerability over time. They may increase emissions, deepen fixed-cost dependence, shift risk to other actors or places, and narrow future adaptation options. In this sense, measures adopted in the name of adaptation may ultimately constitute maladaptation when they temporarily stabilize winter tourism while intensifying longer-term climatic, economic, or institutional vulnerability (7–10).

This study addresses a key gap in the ski tourism literature: how technical “season-saving” measures, under different elevational and regional climatic conditions, evolve into divergent long-term development pathways and risk profiles. Previous studies have generated substantial knowledge on snow reliability, ski-climate sensitivity, and the technical role of snowmaking, while more recent research has shown that Japanese ski resorts increasingly communicate adaptation, mitigation, and year-round diversification strategies in response to climate pressures (6). However, existing work has less often connected changing snow-reliability conditions to the maladaptation risks embedded in policy and investment choices, particularly in contexts where climatic exposure, governance capacity, and operational flexibility vary strongly across regions and resort types (11–14).

To bridge this gap, this study introduces an integrated exploratory screening approach. The SRL concept developed in Alpine research is adapted to the Japanese context as a resort-level exposure framework. Resort policy documents are then examined through Barnett and O'Neill's (7) five maladaptation pathways, extended to consider continuity-driven trajectories alongside action-driven lock-in. This combination links snow-reliability pressure to governance choices and clarifies how similar adaptation strategies can carry different long-term risks across altitude, regional climate, and investment contexts.

Recent comparative studies across ski markets further underline the importance of context. European ski areas show uneven levels of transformational adaptation, while emerging markets such as China reveal distinct patterns in skier perception, resort vitality, and climate response (15–17). These differences suggest that adaptation in ski tourism should not be understood as a uniform response, but as a pathway shaped by climatic exposure, resource conditions, institutional capacity, and development strategy. This is particularly relevant in Japan, where regional climatic contrasts and uneven resort structures create sharply differentiated adaptation conditions.

Accordingly, this study pursues three objectives. First, drawing on the Snow Reliability Line (SRL) concept established in European Alpine studies (11–13, 18), it develops a transparent Japan-calibrated pathway framework for assessing snow-reliability exposure across elevation bands. Second, it applies the Protect Our Winters Japan dataset to examine how technology-governance combinations align with climate-risk exposure (19). Third, it integrates a maladaptation lens with the pathway classification to diagnose where adaptation, mitigation, anticipation, and continuity-oriented development pathways generate lock-in, rising emissions, or delayed structural transition.

## Literature review

### A literature-based framework of snow reliability transition in ski resorts

**Figure 1:**
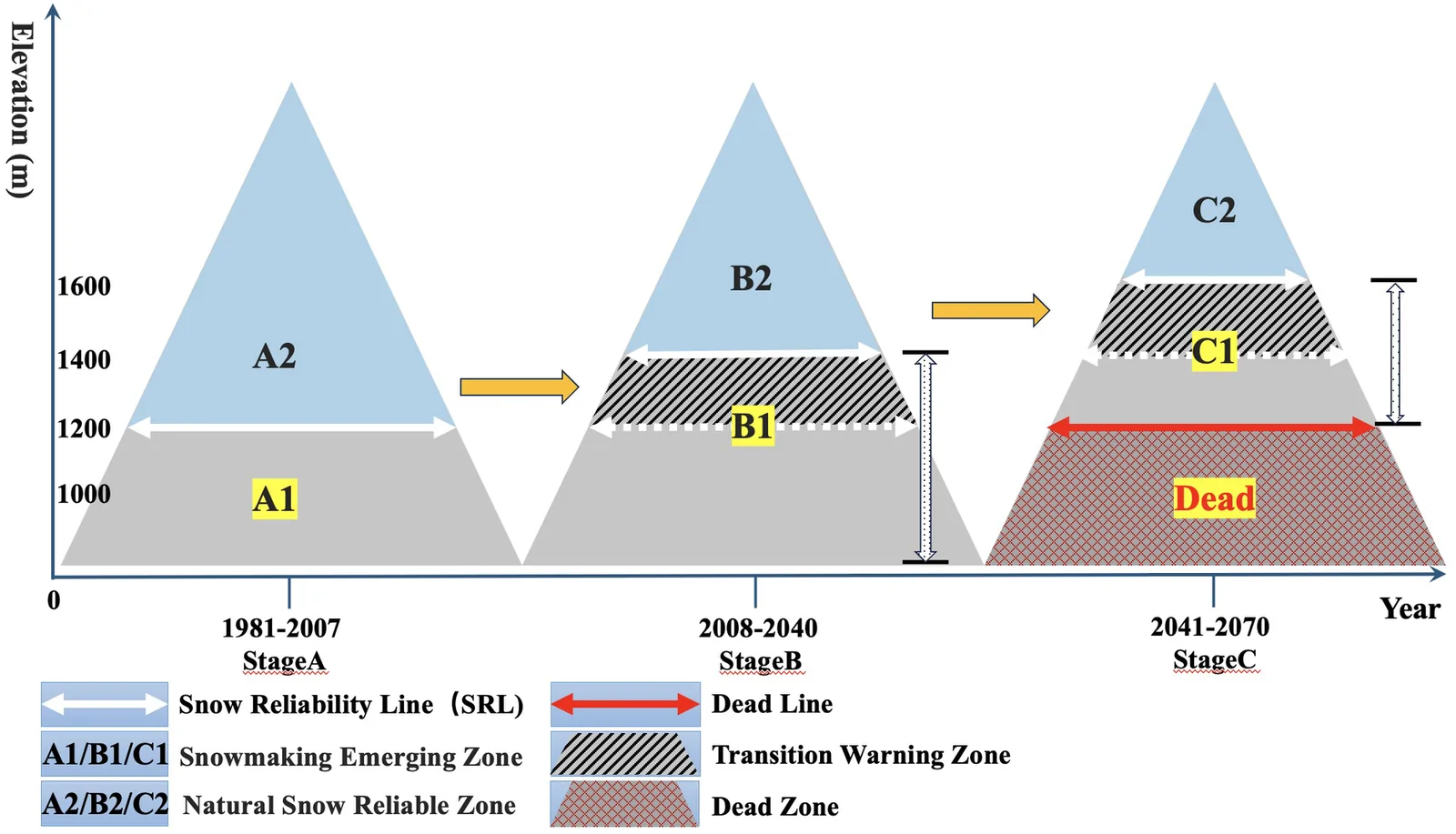
A literature-based framework of snow reliability transition in ski resorts. Source: Compiled by the author from Abegg et al. (2007), Beniston (2003), Marty (2008), Steiger and Abegg (2013), Steiger et al. (2019), Spandre et al. (2019), François et al. (2023), and IPCC (2007, 2014, 2022). The figure is organized into three analytical windows (1981–2007, 2008–2040, and 2041–2070). Within these windows, Stages A–C indicate successive snow reliability conditions associated with the upward shift of the Snow Reliability Line (SRL). The figure distinguishes between naturally snow-reliable zones (A2–B2–C2) and snowmaking-dependent zones (A1–B1–C1), and illustrates the formation of a transition warning zone and a low-elevation dead zone under continued warming.

To conceptualize the sequence of climate-driven transformations, this study draws on and synthesizes multiple key references (2, 3, 5, 9, 11–13, 20–22) to construct the literature-based Ski Resort Snow Reliability Stage Framework (Figure 1). The framework centers on the vertical upward shift of the Snow Reliability Line (SRL), illustrating how the “operationally viable zone” of ski resorts becomes progressively compressed and fragmented under climate warming (11, 13, 14). This figure uses the European Alps as a representative case to illustrate the upward shift of the Snow Reliability Line (SRL) under global warming, a trend consistently confirmed across regional and cross-national studies (11, 13). The figure depicts how rising temperatures drive the vertical relocation of viable ski operating zones, distinguishing between naturally snow-reliable zones (A2 → B2 → C2) and snowmaking-dependent zones (A1 → B1 → C1).

### Upward shift and transition warning zone

While both retain their functional characteristics, the elevations at which they remain operational progressively rise (11, 14). Between successive SRLs, a transition warning zone forms, where natural snow reliability decreases, snowmaking windows shorten, and operational costs increase, although skiing remains technically feasible (13). In Figure 1, these changes are organized into three analytical windows: 1981–2007, 2008–2040, and 2041–2070. These windows are used to facilitate comparison across different warming phases. The first window (1981–2007) corresponds to the historical reference period commonly used to describe baseline snow reliability conditions and ends with the publication of the IPCC Fourth Assessment Report and the OECD synthesis on winter tourism in the Alps (14, 20, 23). The second window (2008–2040) represents an analytical transition period in which declining natural snow reliability, increasing snowmaking dependence, and widening differences between more and less resilient ski areas become increasingly visible (21, 24–26). The third window (2041–2070) corresponds to the mid-century horizon widely used in climate impact assessment, during which snow reliability risk, adaptation limits, and resource constraints become substantially more pronounced under continued warming (3, 14, 22). In the analytical illustration, the SRL is shown at approximately 1,200 m in Stage A, 1,400 m in Stage B, and 1,600 m in Stage C, indicating the broad upward displacement of snow reliability conditions across warming phases.

Regional studies in North America and Canada similarly indicate that while Paris Agreement-aligned pathways lessen snow loss and snowmaking requirements compared to high-emission trajectories, even resilient medium- and large-scale resorts must navigate critical trade-offs among energy, water, and carbon constraints (27, 28). Research has also moved beyond simple elevation-threshold applications of the 100-day rule. Steiger (18) used climate and snowmaking simulations to assess ski-season length and snowmaking requirements in Tyrol, while Spandre et al. (13) developed a spatially explicit snow-reliability assessment using snowpack modelling, climate projections, and ski-infrastructure elevation indicators. These studies show that SRL-based approaches are useful for comparative screening, and that detailed impact assessment benefits from explicit snow-condition modelling, local infrastructure data, and different reliability requirements across ski areas.

### Low-elevation dead zone

Below the lower snow-reliability boundary, a dead zone emerges, where unstable snow cover renders even high-efficiency snowmaking insufficient to sustain core-season operations, signaling structural attrition and spatial reconfiguration among low- and mid-altitude ski resorts (3, 9, 11, 15, 29).

Global-scale projections further suggest that under high-emission scenarios, approximately 13% of existing ski areas may lose all natural snow days by the end of the century, driving resort relocation to higher altitudes or inland regions and generating new ecological and social pressures (30). Recent studies also show that warming reduces the number of hours with wet-bulb temperatures below snowmaking thresholds, thereby narrowing the feasible window for artificial snow production and intensifying trade-offs among water, energy, and carbon emissions (3, 10, 28). Under such conditions, a band of resorts may remain technically operable yet become increasingly marginal in climatic, economic, or environmental terms. This vertical stratification signals an impending spatial restructuring of the ski industry, as many mid- to low-elevation resorts approach economic or biophysical operational limits.

### Scenarios and thresholds

Figure 1 employs a time–elevation analytical structure organized around three analytical windows—1981–2007, 2008–2040, and 2041–2070—to depict the shifting reliability of ski operations across elevation bands. These windows are intended as heuristic stages for comparative interpretation. In the broader ski-climate literature, higher warming benchmarks, including +2 °C conditions, are often used to illustrate the upward contraction of reliable snow zones and the growing limits of snowmaking in marginal areas (2, 3, 11). As warming intensifies toward mid-century, the feasibility of snowmaking becomes increasingly constrained by shorter production windows, resource demands, and rising operating costs. Under higher warming conditions discussed in the literature, reliable snow zones contract upward, mid-elevation resorts become more dependent on snowmaking, and some lower-elevation areas move beyond viable operating limits, reinforcing the need for region-specific interpretation of elevation bands (3, 27, 28).

Overall, warming reduces natural snow cover and compresses the operational window for snowmaking, pushing some parts of the ski industry toward structural limits. Figure 1 provides the conceptual logic of snow reliability transition across elevation bands, while the empirical classification of Japanese ski resorts is developed later in the Methods section through regional calibration, sensitivity checking, and pathway coding.

### Maladaptation

The term maladaptation originates from ecology and evolutionary biology, referring to behaviors or traits that appear advantageous for survival in the short term but result in negative consequences when examined across longer temporal scales or broader system contexts (31).

Since the 2000s, the concept has been incorporated into climate change research to highlight how some nominal adaptation measures may unintentionally increase systemic vulnerability or shift risks to other groups and regions. Barnett and O'Neill (7) developed maladaptation into a policy analysis framework, arguing that an adaptation measure constitutes maladaptation when it reduces risk for one community, sector, or region while increasing risks for others.

Five primary pathways were identified: (1) increased greenhouse gas emissions, where energy-intensive “adaptation” creates positive feedback by pushing risk forward in time; (2) disproportionate burden on the most vulnerable, shifting costs or harms onto low-income, minority, or local groups and producing distributional imbalances; (3) high opportunity costs, diverting resources from more effective alternatives and undermining long-term resilience; (4) reduced incentives to adapt, creating dependency or crowding-out effects that suppress ongoing adaptation and conservation behaviors; and (5) path dependence, where long-lived infrastructure and sunk costs narrow future option sets and limit flexibility under climatic and socio-economic uncertainty.

Applied to the ski industry, these pathways take the following concrete forms. First, expanding snowmaking coverage increases greenhouse gas emissions, as substantial water and electricity inputs elevate carbon footprints, particularly in regions reliant on high-carbon electricity grids (3). Second, resource allocation patterns often burden vulnerable groups: high-altitude and large-scale resorts benefit from capital, technical capacity, and policy support, while low-altitude resorts and dependent communities experience industry contraction and heightened economic fragility (27, 32). Third, long-lived, snow-dependent infrastructure investments can crowd out multi-season diversification and nature-based strategies with greater long-term resilience (32, 33). Fourth, subsidies that stabilize snowmaking may weaken incentives for low-carbon transition, constraining adaptation within existing operational models (10). Fifth, repeated cycles of snow preservation and infrastructure expansion increase asset specificity and reduce strategic and policy flexibility, reinforcing institutional, technological, and financial lock-in (3, 30). Taken together, maladaptation in the ski sector reflects intertwined dynamics of energy dependency, distributive inequity, and governance legitimacy.

This framework emphasizes the unintended side effects of policy and technological choices and the cross-scale transfer of vulnerabilities, forming a theoretical foundation later adopted in the Fifth and Sixth Assessment Reports (AR5 and AR6) of the Intergovernmental Panel on Climate Change (IPCC). Subsequent research has further elaborated on maladaptation from governance and equity perspectives.

Juhola et al. (8) conceptualized maladaptation as both an outcome and an ongoing process, categorizing it into rebounding vulnerability, shifting vulnerability, and eroding sustainable development. This perspective underscores the distributive effects and cross-scale accountability issues inherent in maladaptation, highlighting who benefits and who is disadvantaged through adaptation actions.

A large-scale study of 2,234 ski resorts across 28 countries indicated that under 2 °C and 4 °C warming scenarios, 53% and 98% of resorts, respectively, face very high operational risk without snowmaking; even with 50% snowmaking coverage, risk levels only fall to 27% and 71%, while water and electricity demand and associated carbon emissions increase concurrently (3). These patterns align with maladaptation pathways of increased emissions, high opportunity costs, and path dependence.

Recent review research found that where snowmaking expansion occurs within high-carbon electricity systems, limited water supplies, or subsidy-dependent institutional contexts, it tended to undermine the momentum of low-carbon and multi-season transitions, leading to cumulative costs and emissions indicative of maladaptation; however, these risks could be partially mitigated under low-carbon energy systems, strong water governance, and clear transition planning (10). On the demand side, deteriorating snow conditions reduced tourism demand, though pricing and experience design can offer partial compensation; when multiple regions were affected simultaneously, overall demand declines sharply (33).

Recent studies emphasized that maladaptation often arises from lock-in effects embedded in policy and technological systems, driven by interactions among investment structures, technological dependencies, and institutional inertia (9, 34). Under such conditions, adaptation measures may initially appear reasonable, but if they fail to adjust in response to evolving climate risks, they risk reinforcing existing vulnerabilities. Kates et al. (31) similarly argue that in the context of high climate uncertainty, incremental adjustments are insufficient; without a shift toward transformational adaptation, systems may become locked into irreversible maladaptive trajectories.

### Maladaptation in ski tourism

In tourism and leisure research, the concept of maladaptation has been widely applied to evaluate whether industry strategies and governance approaches achieve long-term resilience (14, 35, 36). The ski resort industry relies heavily on artificial snowmaking, energy subsidies, and long-distance tourist travel to sustain operations, reinforcing a lock-in pathway characterized by high carbon emissions, technological dependence, and demand-driven growth. While these practices maintain short-term profitability and landscape usability, they undermine energy transition feasibility, increase environmental burdens, and erode natural capital regeneration, constituting a typical case of technological maladaptation.

Maladaptation thus occurs when adaptation measures appear to reduce risks in the short term yet simultaneously increase long-term emissions, exacerbate social inequalities, crowd out more forward-looking alternatives, weaken adaptive capacity, or lock systems into inflexible development pathways (7). In the ski industry, this is most evident in the continued reliance on artificial snowmaking, infrastructure expansion, and snow-season extension to sustain operations. While these measures may temporarily stabilize skiable conditions and revenue, they increase energy and water consumption, reinforce rigid cost structures, and impose adverse distributive impacts on neighboring communities and low-altitude resorts. Recent cross-regional quantitative studies show that even when snowmaking coverage increases to 25%–75%, snow supply risks rise significantly across European mountain regions under global warming, accompanied by growth in water and electricity demand and related carbon emissions, revealing an inherent“risk reduction–emissions increase–resource stress “tension characteristic of maladaptive dynamics (3).

The perceptions of different stakeholders—tourists, local communities, and business representatives—toward adaptation strategies are important for their successful implementation (37). Moreover, skiers' perception of climate change, influenced by their activity involvement and place loyalty, plays a significant role in shaping demand-side adaptation behaviors (17).

In addition, multi-regional research in Europe reveals marked spatial disparities in climate risk: high-altitude and capital-intensive resorts sustain competitiveness through technological adaptation, while low-altitude resorts and dependent communities face economic decline, ecological degradation, and employment instability (3). These outcomes correspond to Barnett and O'Neill's pathways of burdening vulnerable groups and incurring high opportunity costs, illustrating how adaptation strategies may reinforce existing inequalities.

Furthermore, as environmental, social, and governance (ESG) and sustainability governance discourse gains prominence, some ski resort corporations employ promotional sustainability narratives to enhance their public legitimacy without implementing substantive structural transformations to their operational models, resulting in practices of greenwashing and strategic legitimacy management (38). Such superficial or deferred adaptation strategies not only fail to reduce climate risks but also delay the transition toward low-carbon operations and year-round diversification, thereby reinforcing path dependence and weakening incentives for sustained adaptation. In broader competitive landscapes, resilient high-altitude destinations may gain advantage, yet increased dependency on winter-based economies without diversification may generate new forms of path dependence (32).

Accordingly, this study employs Barnett and O'Neill's (7) five-pathway framework as its core analytical lens, integrating evidence from tourism and climate policy research to examine how policy and technological configurations generate, reinforce, or mitigate maladaptation risks at the sectoral scale. The analysis treats maladaptation as a pathway issue rather than a property of single measures alone. Under changing climatic conditions, continuity-oriented strategies, business-as-usual investment, and delayed transition can also become maladaptive when they maintain snow-dependent exposure, defer mitigation, or narrow future choices.

## Methods

This study adopted a document-based policy analysis combined with framework calibration, sensitivity checking, and qualitative coding to examine snow-reliability exposure and potential maladaptation risks in Japanese ski resorts under climate change. The research design comprised four stages: (1) data collection and verification, (2) calibration of a Japan-oriented SRL pathway framework, (3) sensitivity checks for elevation representation and regional correction, and (4) policy-text coding and cross-pathway comparison based on a maladaptation framework.

### Data collection

This study uses the Sustainable Resort Alliance dataset released by Protect Our Winters Japan (POW Japan) as its primary sampling frame (19). The analytical sample comprises 23 ski resorts for which resort names, geographic location, summit and base elevations, representative elevation, regional classification, sustainability indicators, and publicly available policy or operational texts could be cross-checked. Recent sector sources report several hundred operating ski areas in Japan: a 2025 policy report records 481 ski areas in 2019, while Japan Funicular Transport Association reporting indicates 417 operating ski resorts in 2025 (39, 40). Against this national context, the 23-resort POW Japan sample represents a small, sustainability-oriented subset rather than the Japanese ski industry as a whole. POW Japan members provide a theoretically relevant sample for examining how sustainability-communicating resorts frame adaptation, mitigation, and operational continuity.

### Ski resort labelling (JP01–JP23)

To enhance figure readability and conserve space, the 23 ski resorts were labeled JP01-JP23. The labeling scheme follows the regional order in the study's baseline roster and is used for indexing, cross-referencing, and figure display. All statistics and graphics use the ski resort as the unit of analysis. A concordance between the labels and the official resort names is available from the corresponding author upon request.

### Japan-Calibrated SRL framework and pathway classification

Building on the literature-based framework presented in Figure 1, this study operationalizes the SRL framework for empirical application in Japan. In this study, the SRL refers to the approximate elevation above which ski resorts are more likely to maintain reliable seasonal operation under a given set of climatic assumptions. The snowmaking buffer (*Δ*) represents the vertical band below the SRL within which snowmaking may partly compensate for declining natural snow reliability. These parameters provide a transparent classification that can be compared with publicly documented resort actions.

### Concept and baseline thresholds

European empirical studies provide the basis for the baseline SRL parameters used in this study. The 100-day rule conventionally defines a ski area as snow reliable when sufficient snow cover, commonly operationalized as at least 30 cm, is available for at least 100 days in a season (11, 13, 18). Earlier SRL applications examined how reliability lines shift upward with warming, while more recent studies have shown that snowmaking, grooming, wet-bulb temperature, local infrastructure, and probabilistic climate conditions shape the operational meaning of snow reliability (3, 13, 18).

Following this literature, the conceptual framework in Figure 1 illustrates baseline SRL thresholds of 1,200 m, 1,400 m, and 1,600 m for Stages A, B, and C. For empirical screening in Japan, these thresholds were recalibrated to 1,250 m, 1,450 m, and 1,650 m. The 50 m upward adjustment aligns the Alpine-derived thresholds with the study's use of resort mean elevation and with Japan's lower-latitude setting, while retaining a simple comparative structure across resorts.

The adjustment was based on three considerations. First, this study uses the arithmetic mean of summit and base elevations as a representative resort elevation because complete ski-lift-power or slope-surface data were unavailable for all resorts. This midpoint proxy differs from lift-power-weighted or slope-area-weighted elevation indicators used in more spatially explicit research (13). Second, Japan's ski regions span a strong north-south gradient, making direct transfer of Alpine thresholds inappropriate without regional interpretation. Third, the pathway framework is designed to compare relative exposure across resorts before linking that exposure to public policy texts.

### Regional zoning and elevation correction

Because Japan spans a pronounced north-south climatic gradient, a single national elevation threshold cannot adequately represent snow-reliability conditions across all ski regions. To address this issue while retaining a unified analytical structure, the study introduces a regional elevation correction (*Δ*z) to account for broad climatic differences.

Based on topographic and climatological characteristics, Japanese ski regions were grouped into three zones: Zone H, covering Hokkaido and northern Tohoku; Zone J, covering central Japan, including Nagano, Niigata, Hokuriku, and the Japanese Alps; and Zone W, covering Kinki and western Japan. Zone H is characterized by colder winters and heavier snowfall, whereas Zone W is comparatively warmer and more climatically marginal for snow-based operations.

The representative elevation of each ski resort was defined as the arithmetic mean of summit and base elevations. The corrected elevation, ℎ′, was then calculated as: ℎ′=Mean Elevation + *Δ*z，where *Δ*z =  + 300 m for Zone H, 0 m for Zone J, and −200 m for Zone W.

Table 1 presents Japan Meteorological Agency (JMA) 1991–2020 climate normals for five representative stations across these zones (41). The December-February (DJF) mean temperature ranges from −2.3 °C at Sapporo (Zone H) to 6.4 °C at Hiroshima (Zone W), yielding an inter-zone temperature difference of approximately 8.7 °C. Annual snowfall ranges from 479 to 567 cm in Zone H to 8 cm in Zone W, and maximum snow depth ranges from 95 cm at Sapporo to 23 cm at Niigata where recorded. These data document a pronounced winter climate gradient consistent with the three-zone structure.

**Table 1:** JMA 1991-2020 winter climate normals f1or representative stations.

Zone	Representative station	Station elevation (m)	DJF mean temperature (C)	Annual snowfall (cm)	Maximum snow depth (cm)
H	Sapporo (Hokkaido)	17	−2.3	479	95
H	Aomori (Aomori)	3	−0.1	567	111
J	Nagano (Nagano)	418	0.9	263	34
J	Niigata (Niigata)	4	3.9	217	23
W	Hiroshima (Hiroshima)	53	6.4	8	-

Source: Japan Meteorological Agency 1991-2020 climate normals and past weather data search (Japan Meteorological Agency, 2024).

DJF, December-January-February. Station elevations are JMA observatory elevations, not ski resort elevations. The table documents regional climatic contrasts for screening calibration, not resort-level snowpack conditions.

Applying the documented snowline sensitivity of approximately 150 m per +1 °C (11, 26), the observed inter-zone temperature difference of 6.4–8.7 °C translates to an expected SRL elevation differential of roughly 960–1,305 m between Zones H and W. For the present exploratory screening framework, a narrower correction range is adopted: *Δ*z =  + 300 m for Zone H, 0 m for Zone J, and −200 m for Zone W. This conservative correction reflects the fact that city-station temperatures do not directly represent resort-elevation lapse rates, snowfall amount also contributes to operational snow reliability, and the *Δ*z correction is designed for first-order screening rather than high-resolution climate mapping. Adopting the full 960–1,305 m range would over-correct for Zone H and over-penalize Zone W, because the city-station temperature gradient steepens at low elevations and does not scale linearly to resort altitudes. The 500 m correction range reflects a conservative assumption that the effective resort-elevation temperature gradient is approximately half of the city-level gradient.

### Resort classification and pathway coding

To classify snow-reliability exposure, the study combines the Japan-calibrated SRL thresholds with a standard snowmaking buffer (*Δ* = 200 m). This value is consistent with the 150–200 m buffer range discussed in earlier SRL and snowmaking studies (11, 12, 26). Recent snowpack-modelling studies further show that snowmaking feasibility depends on wet-bulb temperature, water availability, grooming, operating standards, and investment capacity (3, 13, 28). The 200 m buffer is used here to distinguish broad screening classes in a transparent and reproducible way. The probabilistic snow-reliability framework of Spandre et al. (13), which requires spatially explicit snowpack modelling (SAFRAN–Crocus) not yet operationalized for Japanese ski areas, represents a more advanced approach for future research; the present deterministic buffer is used as a screening precursor to such modelling.

Using corrected elevation ℎ′, resorts were classified at each stage according to whether they fell above the relevant SRL threshold, within the snowmaking buffer below that threshold, or below the operationally viable range. Stage A, Stage B, and Stage C correspond to threshold values of 1,250 m, 1,450 m, and 1,650 m, respectively. Resorts above the threshold were coded as naturally snow-reliable (A2, B2, C2), whereas those located within 200 m below the threshold were coded as snowmaking-dependent (A1, B1, C1).

The final snow reliability status for each resort was expressed in an A-B-C trajectory format, such as A1-B1-C1 or A2-B2-C2. This pathway format captures how a resort's relative snow reliability position changes across successive analytical windows and allows direct comparison between more stable, transitional, and highly marginal operating conditions. In this way, the pathway classification operationalizes the conceptual logic of Figure 1 in a form adapted to the Japanese context.

### Operational lower bound (deadline rule)

To distinguish highly marginal resorts from those that remain technically operable through snowmaking, the study introduces an operational lower bound termed the Japan SRL-Deadline. The value of 1,000 m identifies resorts whose corrected elevation places them below the assumed operationally viable band in the final analytical window. This category marks long-term exposure to climatic and operational pressure in the pathway classification.

When corrected elevation h′ is lower than 1,000 m, the resort is coded as entering the Dead category in Stage C. This rule combines climatic feasibility, seasonal reliability, operating cost, and peak-period availability when evaluating long-term ski operations.

The deadline rule aligns the pathway classification with long-term operational sustainability. It focuses the typology on resorts that can sustain core-season service and peak-period availability under changing climatic and economic conditions. Table 2 summarizes the Japan SRL-calibrated operational classes by corrected elevation.

**Table 2:** Japan SRL-calibrated operational classes by corrected elevation (h′).

Corrected elevation h′ (m)	Operational class	Screening interpretation
h′ < 1,000	Dead/operational lower-bound breach	Below the Japan SRL-Deadline; long-term core-season operation is unlikely even with snowmaking under the screening assumptions.
1,000 <= h′ < 1,250	Marginal/snowmaking-dependent	Shorter and less reliable snow season; high dependence on snowmaking and elevated risk during peak holiday periods.
1,250 <= h′ < 1,450	Feasible/transition zone	Potentially operable under current screening assumptions, but exposed to rising snowmaking needs and operating-cost pressure.
h′ >= 1,450	Highly reliable/naturally advantaged	Longer and more consistent snow season; lower relative climatic exposure within the sample.

Source: Compiled by this study from Abegg et al. (2007), Steiger & Abegg (2013), the Japan-calibrated SRL thresholds (1,250/1,450/1,650 m), and the Japan SRL-Deadline (1,000 m).

### Parameter choice and classification output

This study adopts a snowmaking buffer of *Δ* = 200 m for the baseline classification. Based on corrected mean elevation, SRL thresholds, and the operational lower bound, five snow-reliability pathways were identified: A1-B1-Dead, A1-B1-C1, A2-B1-C1, A2-B2-C1, and A2-B2-C2. These pathways provide the basis for subsequent comparison with maladaptation coding.

### Sensitivity analysis

To assess the influence of key parametric assumptions on pathway classification, four sets of sensitivity checks were conducted on the 23-resort sample.

First, the SRL thresholds were varied by ±25 m, to 1,225/1,425/1,625 m and 1,275/1,475/1,675 m. No resort changed pathway under either variant, indicating that the precise value of the 50 m Japan calibration is not a critical sensitivity parameter within this range.

Second, the snowmaking buffer *Δ* was varied to 150 m and 250 m. No resort changed pathway under either variant in the 23-resort sample.

Third, the regional correction *Δ*z was varied across three alternative scenarios: compressed correction (+200/+100/−300 m for Zones H/J/W), expanded correction (+400/−100/−100 m), and no correction (0 m for all zones). Under the compressed scenario, 7 of 23 resorts (30.4%) changed pathway, predominantly shifting upward (A1 to A2 or A2-B1-C1 to A2-B2-C1). Under the expanded scenario, 4 resorts (17.4%) changed pathway. Under no correction, 2 Zone H resorts (8.7%) shifted downward from A1-B1-C1 to A1-B1-Dead when their regional climatic advantage was removed.

Fourth, the representative elevation indicator was varied from mean elevation to base elevation and summit elevation. Base elevation produced the largest single-parameter effect: 13 of 23 resorts (56.5%) shifted toward more marginal categories. Summit elevation shifted 14 resorts (60.9%) toward more stable categories. The baseline mean-elevation classification thus occupies a midpoint position with known directional bias.

Finally, combined worst-case (base elevation, no *Δ*z, *Δ* = 150 m, SRL +25 m) and best-case (summit elevation, baseline *Δ*z, *Δ* = 250 m, SRL −25 m) scenarios were constructed to bound the plausible classification range. Under the worst-case scenario, 16 of 23 resorts classified as A1-B1-Dead; under the best-case scenario, 9 resorts classified as A2-B2-C2. Neither extreme represents operational reality; together they define the envelope within which the true classification is expected to lie.

Across all sensitivity tests, 6 of 23 resorts (26.1%) retained the same pathway classification, while 17 (73.9%) changed under at least one variant. These results are consistent with the study's use of the SRL pathway as a comparative screening typology, designed to organize resorts into relative exposure categories for cross-referencing with policy texts, rather than as a high-resolution operational forecast.

### Policy text coding and comparison of maladaptation pathways

#### Analytical framework

Building upon the pathway classification developed above and Barnett and O'Neill's (7) framework, this study evaluates whether resort development and operational strategies mitigate short-term risks while introducing new or longer-term vulnerabilities. The analysis includes visible adaptation measures, mitigation-oriented measures, anticipatory transition measures, and continuity-oriented business-as-usual strategies. This broader scope captures maladaptation as a development pathway shaped by technical choices, investment priorities, and delayed transition.

Drawing on recent research in the ski tourism sector (e.g., 3, 9, 10, 26), resort actions are categorized into five potential maladaptive pathways: (1) increasing greenhouse gas emissions, (2) amplifying burdens on vulnerable groups, (3) incurring high opportunity costs, (4) undermining incentives for sustained adaptation, and (5) reinforcing path dependence. Mitigation and anticipation measures were used to evaluate whether an apparently adaptive action was accompanied by emissions reduction, transition planning, or investment flexibility.

#### Coding procedure

The unit of analysis is the individual ski resort, each corresponding to a single SRL pathway but potentially coded under multiple maladaptation (MA) categories. Policy-related text segments were extracted from official and publicly available documents, including resort homepages, sustainability pages, facility descriptions, ticketing and access information, event announcements, infrastructure updates, and governance or certification statements. Each segment was coded when the text provided an observable action, investment, service design, or governance indicator linked to one of the decision rules in Table 3. Multiple policy actions at the same resort within the same MA category were treated as a logical union, so a resort was counted at most once in each MA x Path cell.

**Table 3:** Criteria for maladaptation (MA1-MA5): decision rules.

MA	Criterion	Operational decision rule
MA1	Increasing greenhouse gas emissions	Code when a documented policy expands winter visitor-hours, operating time, facility intensity, or transport activity likely to increase aggregate energy use or emissions, even if efficiency improves.
MA2	Amplifying burdens on vulnerable groups	Code only when public documents provide evidence that costs or risks are shifted to local communities, low-income residents, seasonal workers, or other vulnerable groups.
MA3	High opportunity costs	Code when texts or indicators show resources, attention, or investment being directed to image-, certification-, or experience-oriented actions in ways that substitute for structural transition.
MA4	Undermining incentives for sustained adaptation	Code when labels, registrations, awards, or substitute indicators are used in place of ongoing adaptation efforts or crowd out long-term transition investment.
MA5	Reinforcing path dependence	Code when fixed assets, engineering works, winter-only services, or institutionalized destination narratives deepen commitment to the snow-season business model.

Source: Compiled by this study from Barnett and O'Neill (2010), Juhola et al. (2016), Scott et al. (2024), François et al. (2023), and the coding procedure described above.

The coding followed conservative evidentiary rules. MA1 required evidence of expanded visitor-hours, operating time, transport activity, facility intensity, or winter commerce likely to increase aggregate energy use or emissions. MA3 and MA4 required textual or indicator-based evidence that image-oriented, certification-oriented, or experience-oriented initiatives substituted for structural transition. MA5 required evidence of fixed assets, institutionalized winter services, or engineering-based extensions that increased commitment to the snow-season model. Cases without clear textual evidence were left uncoded, even when the resort occupied a high-risk SRL pathway.

### Coding example: JP10 (Niseko Tokyu Grand Hirafu)

To illustrate the coding procedure, this section presents the evidentiary trail for JP10, an A1-B1-C1 resort in Zone H with corrected elevation h′ = 1,030 m. Three publicly available documents were analyzed.

First, a news announcement describing the opening of HIRAFU MARCHE, a winter retail facility framed within a 50th-anniversary ski-town vision, was coded under MA1 because the new facility increases on-site activity and visitor dwell time, under MA3 because the investment reinforced winter branding without documented parallel four-season transition, and under MA5 because the retail space and ski-town narrative constitute fixed assets and institutionalized winter identity.

Second, a sustainability statement declaring completion of a 100% renewable-energy transition for resort operations was retained as mitigation evidence. Because the statement did not address demand-side growth or transport emissions, it was not coded as MA1, MA3, or MA4. MA4 was considered but not coded under the conservative evidentiary standard because the available text did not show that the renewable-energy claim substituted for, rather than complemented, ongoing adaptation efforts.

Third, a spring-season announcement promoting Golden Week skiing with discount tickets and gondola access was coded under MA1 because extended operating days and stimulated visitation increase aggregate energy use, under MA3 because discount promotions and gondola access prioritize winter visitor volume over structural diversification, and under MA5 because gondola infrastructure extends the snow-dependent operating model.

After applying the logical union rule, JP10 was coded as MA1 + MA3 + MA5 in the A1-B1-C1 pathway. The renewable-energy statement was recorded as mitigation context but was not used by itself to assign MA4. Table 4 summarizes the complete coding trace.

**Table 4:** JP10 coding trace across three public documents.

Document/public text	Evidence used for coding	MA1	MA2	MA3	MA4	MA5
HIRAFU MARCHE and 50th-anniversary ski-town narrative	Winter retail expansion increased on-site activity, visitor dwell time, and winter branding; no parallel four-season or mobility transition was documented.	Coded	Not coded	Coded	Not coded	Coded
100% renewable-energy transition statement	Recorded as mitigation evidence. It reduced the basis for MA1 in this document, but did not address demand-side growth or transport emissions.	Not coded	Not coded	Not coded	Considered but not coded: no evidence that the claim substituted for ongoing adaptation.	Context only; not sufficient alone
Golden Week spring-skiing promotion and gondola access during construction	Season extension, promotional pricing, and gondola access supported continued snow-season visibility and fixed infrastructure use.	Coded	Not coded	Coded	Not coded	Coded

Source: Compiled by this study from public JP10 resort documents and the MA1-MA5 decision rules in Table 3.

“Not coded” means that the public document did not meet the conservative evidentiary threshold for that MA category. MA4 was considered for the renewable-energy statement but not coded because the document did not show that the claim replaced or crowded out ongoing adaptation efforts.

## Results

### Sample and basic characteristics

This study includes 23 ski resorts drawn from the Protect Our Winters Japan dataset, covering major snow regions such as Hokkaido, Tohoku, Chubu, Kinki, and Western Japan. The representative elevations range from approximately 533 to 2,240 meters, exhibiting distinct vertical and latitudinal gradients. The sample provides a structured basis for comparing SRL pathway exposure with publicly documented resort actions.

### Classification and proportion of operational feasibility

**Figure 2:**
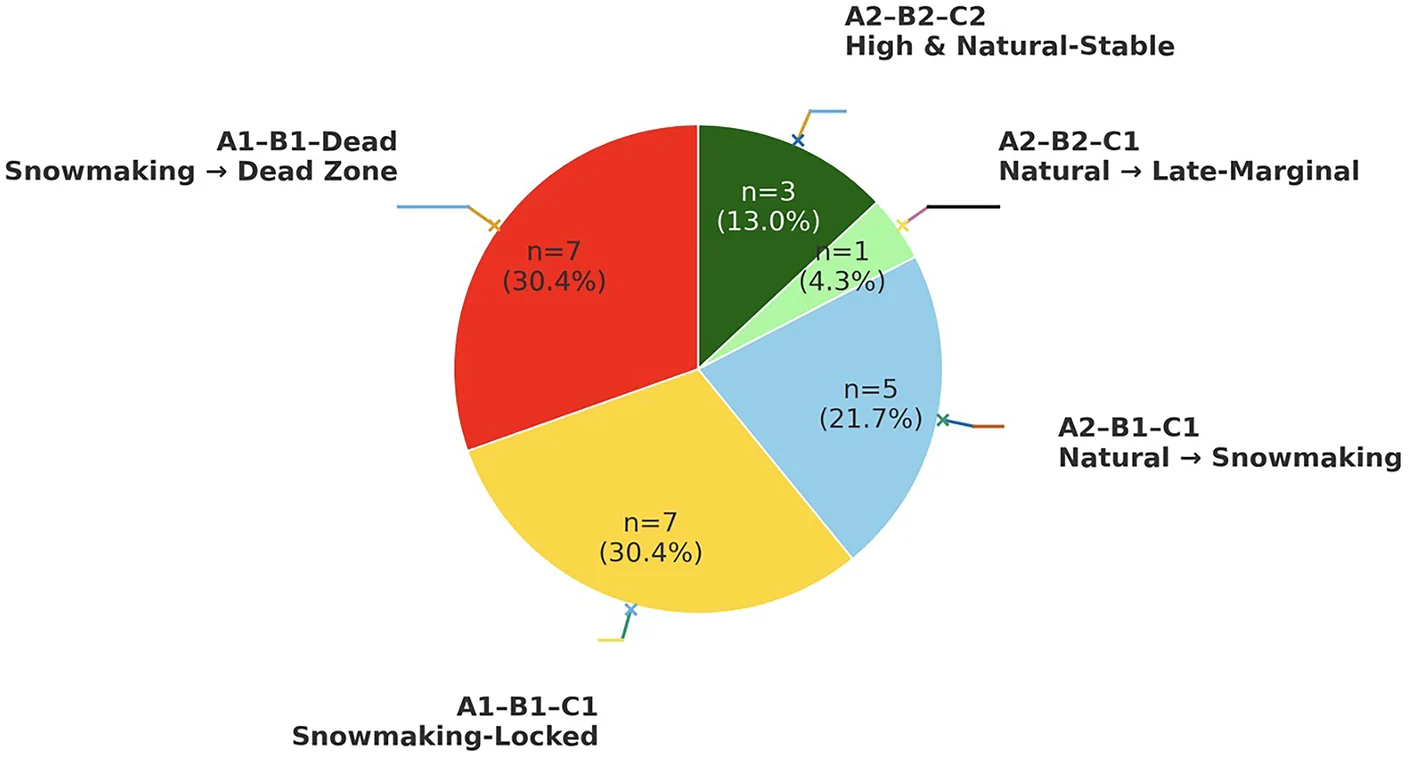
Percentage distribution of ski resort transition pathways. Source: Compiled by this study from the POW Japan resort roster, verified resort elevation data, regional correction assumptions, and the SRL pathway classification described in the Methods section. Percentages are calculated from 23 resorts using corrected mean elevation h′ as the baseline indicator. A1–B1–Dead | Snowmaking → Dead Zone: already within the snowmaking band in A and B and dropping into the SRL dead zone by C (operations unlikely even with snowmaking). A1–B1–C1 | Snowmaking-Locked: snowmaking required in all three windows, indicating a high risk of technological lock-in. A2–B1–C1 | Natural → Snowmaking: naturally reliable in A but entering the snowmaking band in B and C, implying declining reliability and rising costs. A2–B2–C1 | Natural → Late-Marginal: naturally reliable through A and B, becoming snowmaking-dependent only in C. A2–B2–C2 | High and Natural-Stable: remaining above the SRL in all three windows, i.e., the least exposed group.

Following the regional calibration procedure established for Japan and applying the three-tier SRL thresholds, the 23 ski resorts were classified into five operational pathways (Figure 2): A1-B1-Dead (7 sites, 30.4%), A1-B1-C1 (7 sites, 30.4%), A2-B1-C1 (5 sites, 21.7%), A2-B2-C1 (1 site, 4.3%), and A2-B2-C2 (3 sites, 13.0%). These percentages are derived from the resort-level classification table using corrected mean elevation h′ as the baseline indicator. Sensitivity checks show that the overall marginality pattern remains similar when the regional correction is removed, while the choice of representative elevation has a stronger influence on individual pathway assignment.

### Policy correspondence results: distribution of maladaptation labels

#### Overall distribution

**Figure 3:**
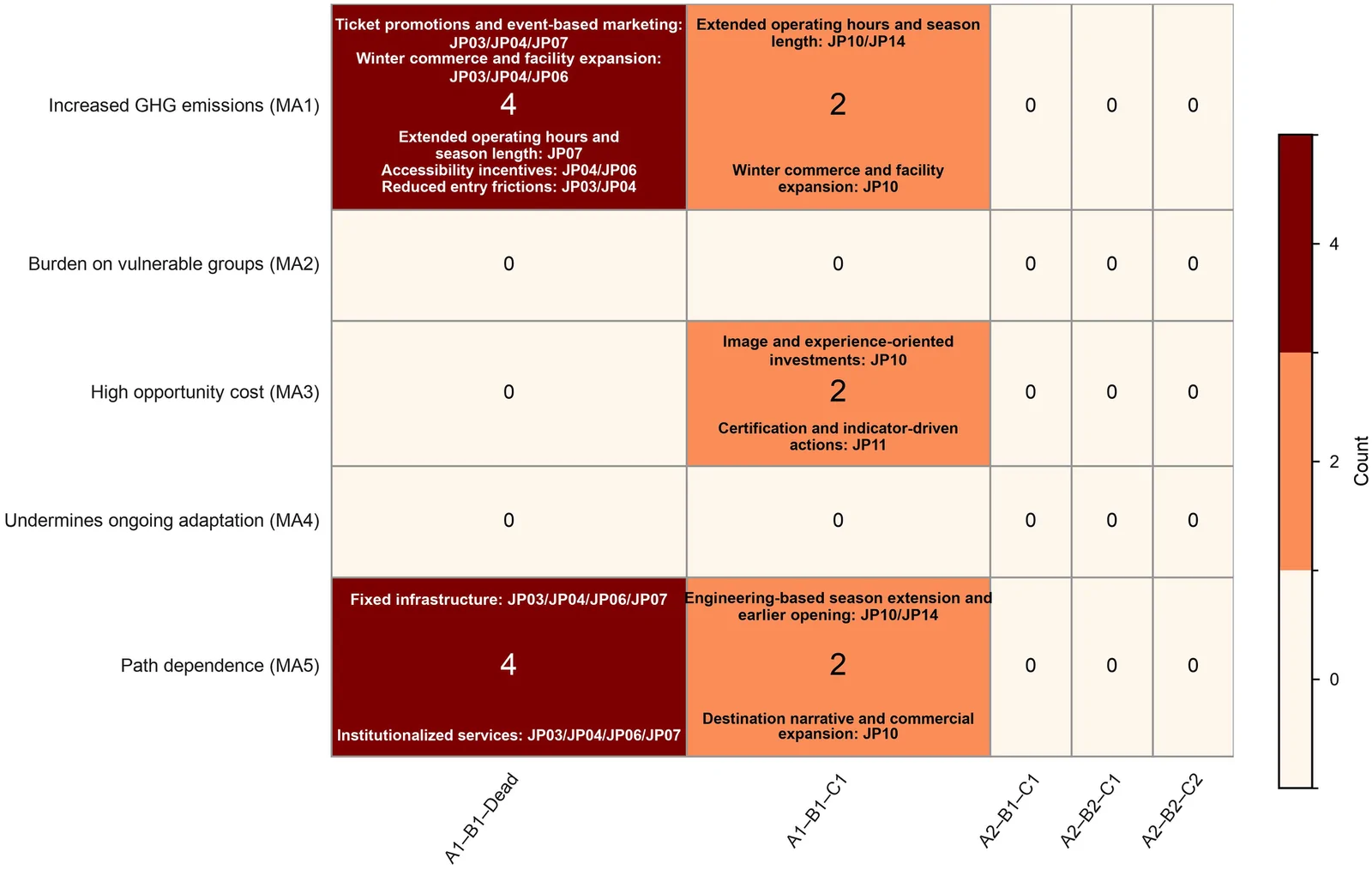
Path × MA heatmap (counts + resort codes). Labels show count and codes. Rows are MA1–MA5 (from top to bottom), and columns are SRL paths (from left to right). Unit: resorts; each resort is counted at most once within the same MA  ×  Path cell. The matrix is based on conservative coding of publicly available resort documents. Zero cells indicate that the available texts did not meet the coding rule for that MA category. The color scale uses the Color Brewer Orange–Red (OrRd) sequential palette; darker shades indicate higher counts.

This study aggregates these annotations into a 5   ×   5 heat map of Path × MA (Figure 3) to examine distributional patterns and concentrations. As summarized in Figure 3, MA1 (increased GHG emissions) and MA5 (path dependence) were most prevalent, concentrated in A1-B1-Dead and A1-B1-C1. MA3 (high opportunity cost) occurred only in A1-B1-C1, where branding- or experience-oriented initiatives substituted for structural transition. By construction, the matrix counts each resort at most once per MA x Path cell; multiple policies at the same resort do not inflate cell counts. In this sample, the counts were: MA1 = 6 (A1-B1-Dead = 4; A1-B1-C1 = 2), MA3 = 2 (A1-B1-C1 = 2), and MA5 = 6 (A1-B1-Dead = 4; A1-B1-C1 = 2); all other cells were zero. Because the coding is based on public documents rather than measured snow, energy, emissions, financial, or transport data, the following results are interpreted as document-based indicators of maladaptation risk rather than direct estimates of environmental impact.

#### MA1 — increased greenhouse gas (GHG) emissions (*n* = 6/23; concentrated in A1–B1–dead = 4, A1–B1–C1 = 2)

This study identifies five document-based clusters of responses: (1) ticket promotions and event-based marketing (JP03, JP04, JP07; *n* = 3); (2) extended operating hours and season length, including later night operations to 22:00, earlier opening, and spring extension through Golden Week (JP07, JP14, JP10; *n* = 3); (3) accessibility incentives, including improved car access and more direct routing (JP06, JP04; *n* = 2); (4) reduced entry friction, including faster gate access and ticketing (JP04, JP03; *n* = 2); and (5) winter commerce and facility expansion (JP03, JP04, JP06, JP10; *n* = 4). Across cases, these texts indicate expanded activity and operating time. Even where per-unit energy use may decline, such expansion indicates higher aggregate-emissions risk unless accompanied by verified energy and transport decarbonization. Representative examples include JP07's extension of night operations to 22:00, JP14's gondola-centered reconfiguration enabling earlier opening, and JP10's spring extension through Golden Week to maintain market visibility and cash flow during gondola construction.

#### Ticket promotions and event-based marketing

JP03 structured its homepage and winter landing pages around a conversion funnel emphasizing ticket types, discounts, and easy access to expand winter demand among entry-level and family segments; JP07 ran multi-wave promotion and publicity campaigns to stimulate near-term and repeat visitation; and JP04 leveraged competitions and events to drive peak-period footfall, complemented by family-oriented content to broaden reach. Collectively, these tactics relied on marketing and price signals to increase both first-time and repeat visitation, thereby increasing winter footfall and operating hours, consistent with MA1's “activity expansion” signal (JP03, JP04, JP07).

#### Extended operating hours and season length

JP07 extended night operations to 22:00, increasing daily operating hours and likely energy demand. JP14 advanced the season opening through engineering interventions and reoriented operations around a new gondola, thereby lengthening the season. Despite ongoing gondola construction, JP10 extended spring operations through Golden Week to maintain market visibility and cash flow. The shared mechanism was temporal extension across daily and seasonal scales. Even where energy intensity declines, longer operating hours indicate higher aggregate energy-demand risk, consistent with MA1's “activity expansion” signal (JP07, JP14, JP10).

#### Accessibility incentives

JP06 coupled free parking and expressway access with family-oriented facilities, including a kids' park, tree runs, rentals, and ski schools, to grow the self-drive family segment. JP04 advertised weekday free parking and emphasized direct rail access and shuttle connections to reduce travel barriers. These accessibility and cost incentives may increase private-car visitation and transport-related emissions risk when they are not paired with demand management or low-carbon mobility measures, aligning with MA1's mobility mechanism (JP06, JP04).

#### Reduced entry frictions

JP04 deployed IC-card gates, automated ticket machines, and advance online purchase options to shorten queues and increase gate-processing efficiency; JP03's page architecture and user flow were likewise optimized to accelerate conversion. Lowering entry friction increased hourly throughput and equipment utilization, amplifying on-site footfall and operating time, consistent with MA1's “activity expansion” signal (JP04, JP03).

#### Winter commerce and facility expansion

JP10 introduced HIRAFU MARCHE and expanded the winter retail footprint as part of a 50th-anniversary“ski town”narrative, which strengthened customer flow and visitor dwell time, and increased electricity use and HVAC loads. Fixed-site services at JP03, JP04, and JP06—children's snow parks, ski schools, and rental operations—although primarily associated with MA5 (path dependence), also contributed to MA1: expansions in spatial extent and service intensity increased footfall and equipment operating time, thereby raising energy use, consistent with MA1's “activity expansion” signal (JP10, JP03, JP04, JP06).

In summary, these behavioral clusters indicate higher activity volumes, longer operating periods, and greater throughput. In the absence of concurrent mobility-side decarbonization (for example, rail-to-shuttle transfers and parking-capacity or pricing management) and deep energy-side decarbonization, tactics such as promotion, temporal extension, enhanced accessibility, and expedited entry may increase aggregate-emissions risk. This interpretation is consistent with MA1's “activity expansion” signal rather than a direct emissions estimate.

#### MA3 — high opportunity cost (*n* = 2/23; A1–B1–C1 = 2)

This study observed two patterns: (1) image and experience-oriented investments—JP10's “ski-town” narrative with winter-retail expansion (HIRAFU MARCHE) that prolonged visitor dwell time and spending; and (2) certification and indicator-driven actions—JP11's prefectural SDGs registration with 2030 targets. The available materials and KPIs showed no parallel plans or investments for structural transition (e.g., transport decarbonization, four-season diversification, or energy-mix shifts). Managerial attention and capital were crowded out by winter branding or indicator compliance; the study therefore coded these cases as MA3.

#### Image and experience-oriented investments (branding, market/retail-circulation expansion)

JP10, through HIRAFU MARCHE and an expanded winter retail footprint under a “ski-town” narrative, increased retail traffic and enhanced experiential offerings, thereby extending visitor dwell time and spending. These investments primarily targeted on-site experience and brand visibility. However, the available materials showed no parallel plans or investments in mobility decarbonization, four-season diversification, or structural transition (e.g., modal shift with demand management, rebalancing toward non-winter products). Because managerial attention and capital were directed toward winter branding and retail activation, there was a risk of crowding out structural transformation.

#### Certification and indicator-driven actions (potential indicator-substitution effects)

JP11 leveraged registration in a prefecture-level “SDGs Promotion Enterprise”program. Its three 2030 targets emphasized reducing single-use items, utilizing local resources, and developing experiential programs. While these actions yielded reputational benefits and local linkages, the visible indicators remained managerial- and marketing-oriented and devoted limited attention to structural issues such as high-carbon mobility, the energy mix, and four-season transition. When certifications and declarations functioned as proxy performance indicators, resources and governance attention were easily diluted.

In summary, the commonality across these two approaches was the preferential allocation of resources to image- and experience-oriented or certification- and indicator-driven improvements, without concurrent planning for structural transition—mobility decarbonization, demand management with four-season diversification, and deep energy decarbonization. Under such conditions, even where brand strength and visitor experience improved in the short term, opportunity costs delayed or crowded out critical transitions, supporting an MA3 determination.

#### MA5 — path dependence (*n* = 6/23; A1–B1–dead = 4, A1–B1–C1 = 2)

This study observed four document-based path-dependence mechanisms, ordered by frequency: (1) fixed infrastructure, including lifts, snow escalators, and zoned terrain (JP14, JP03, JP04, JP06, JP07; 5 of 6 sites); (2) institutionalized services, including school systems, rental systems, and ticketing architecture (JP03, JP04, JP06, JP07; 4 of 6 sites); (3) engineering-based season extension and earlier opening (JP14, JP10; 2 of 6 sites); and (4) destination narratives and commercial expansion, including winter-oriented branding and retail traffic (JP10; 1 of 6 sites). These signals indicate higher asset specificity, stickier labor and capital allocation, and longer daily or seasonal occupancy. Under accelerated warming, they are interpreted as evidence of path-dependence risk rather than direct proof of irreversible lock-in.

#### Fixed infrastructure (lifts/snow escalators/zoned terrain)

At JP14, a gondola-centered expansion, coupled with engineering interventions that advanced the opening date, directed fixed capital toward winter-dependent operations. At JP03, a children's snow park with a snow escalator and zoned beginner- and family-oriented terrain tied the resort more closely to a snow-centric product portfolio. At JP04, the kids' park and permanently zoned family areas similarly anchored fixed capital to winter uses. At JP06, a kids' park and designated tree-run terrain committed infrastructure and layout to snow-dependent operations. At JP07, the kids' park and permanently zoned areas, alongside night-skiing capacity, embedded the winter operating model. As these fixed installations accumulated, flexibility to pivot toward four-season programming or lower-carbon substitutes declined, reinforcing path-dependence risk.

#### Institutionalized services (school system/rental system/ticketing architecture)

At JP03, the resort built a low-friction ticket-and-discount funnel and winter-focused landing pages to convert entry-level and family visitors. This kept gate throughput and process design centered on the winter product and institutionalized a ticketing-led operating model. At JP04, IC-card gates, automated ticket machines, and advance online purchase options accelerated entry, while the children's snow park, ski school, and rental programs professionalized high-throughput winter operations. At JP06, free parking was paired with long-lived family infrastructure, tree runs, rentals, and ski-school systems to grow the self-drive family segment and extend dwell time. At JP07, night-skiing operations to 22:00, alongside a kids' park, ski school, and rentals, captured after-work and local demand. Across these cases, permanent capacity and staffing needs tied labor and capital to winter operations, deepening path-dependence risk.

#### Engineering-based season extension and early opening

JP14 advanced the season opening through engineering interventions paired with a gondola-centered expansion, thereby lengthening the snow season. JP10 extended spring operations through Golden Week during construction of a new gondola to maintain winter revenue and market visibility. Where the season was externally extended through engineering, season length and capital occupancy increased in tandem, likely raising future switching costs for shifts toward low-carbon or four-season operations and reinforcing path-dependence risk.

#### Destination narrative and commercial expansion (winter-oriented branding and retail circulation)

JP10's“ski-town”destination narrative, together with the expansion of winter commercial spaces (e.g., HIRAFU MARCHE), concentrated managerial attention and investment decisions on winter operations. The interaction of branding and retail development intensified dependence on winter products at the governance and spatial levels, representing a canonical MA5 (path dependence) mechanism.

In summary, taken together—fixed capital, institutionalized services, engineering-based extensions, and narrative reinforcement—these trajectories progressively increased lock-in across technological, capital, and governance domains. Under accelerated warming, transition flexibility declined, and both sunk and switching costs were amplified. Accordingly, this study coded these cases as MA5.

#### Continuity as a maladaptive pathway

The coding framework requires observable policy actions, investments, or governance indicators to assign MA codes (Table 3). This design captures active maladaptation, namely cases where specific resort measures increase emissions, create opportunity costs, or deepen path dependence. It may undercount maladaptation arising from inactive continuity, where resorts in high-risk SRL pathways maintain snow-dependent business models without documented transition planning.

Of the 14 resorts classified in the two highest-risk pathways (A1-B1-Dead and A1-B1-C1), 8 show active maladaptive actions coded under MA1, MA3, or MA5. The remaining 6 resorts in these pathways provide no public evidence of transition planning in the materials reviewed: no diversification beyond winter operations, no low-carbon mobility strategy, no phased investment reallocation, and no stated reduction in snow dependency. These cases are treated as potential continuity-driven maladaptive exposure rather than as confirmed intentional inaction.

Under continued warming, as the SRL rises and snowmaking windows narrow, these resorts face escalating operating costs and declining reliability without having built alternative revenue streams or reduced their exposure to snow-conditional income. Continuity in this context is not a neutral baseline: it is an implicit development pathway that defers transition costs to a future with fewer options and higher adjustment burdens.

This finding suggests two implications. First, Barnett and O'Neill's (2010) five pathways, originally designed to classify actions, can be extended to classify inaction or delayed transition when climatic exposure is known and transition options remain available. Second, it highlights a methodological limitation of document-based coding: absence of evidence in public materials may reflect either genuine inaction or unobserved private planning. Future research using interviews, investment data, and longitudinal resort records should test this continuity pathway directly.

## Discussion

The absence of MA2 and MA4 in Figure 3 indicates non-detection under the present evidentiary rules; it does not show that these pathways are absent from the Japanese ski sector. MA2 concerns burdens on vulnerable groups, but such distributive effects are rarely reported in public corporate materials and would require evidence on workers, communities, local public finance, or resource allocation that was not available in the reviewed documents. MA4 requires evidence that a sustainability or mitigation initiative displaced, weakened, or delayed ongoing adaptation. Mere coexistence with adaptation is insufficient for coding. For example, the JP10 renewable-energy statement was considered but not coded as MA4 because the available text did not demonstrate such substitution. The zero counts may therefore reflect conservative coding and disclosure constraints, a sector-specific pattern, or both; the present data cannot distinguish among these explanations.

The sustainability orientation of the 23-resort POW Japan sample also shapes interpretation. The presence of MA1 and MA5 risks among resorts that actively communicate sustainability suggests that climate awareness alone does not prevent maladaptation. Structural features of ski tourism, including fixed winter infrastructure, snow-dependent revenue, and growth-oriented operating strategies, may continue to generate risk. This makes the sample analytically informative, but it does not establish the prevalence of these risks across the wider Japanese ski industry. Resorts with less visible climate engagement may face equal or greater exposure, but they may also disclose fewer actions, making document-based coding more difficult. Accordingly, the findings offer an exploratory warning and do not provide a sector-wide prevalence estimate. A nationally stratified sample combining public documents with interviews, energy-use records, investment data, and community-level evidence is needed to test whether the observed MA1 and MA5 patterns extend beyond sustainability-oriented operators.

### Practical implications

#### Year-round greenhouse gas accounting and emission reduction

This study shifts the analytical focus from activity expansion toward carbon-emission governance, recommending that ski resorts establish year-round greenhouse gas (GHG) accounting frameworks. The pathway analysis (Figure 3) shows that maladaptation risks, especially MA1 (increased GHG emissions) and MA5 (path dependence), concentrate in two snowmaking-dependent trajectories: A1-B1-Dead and A1-B1-C1. Low-elevation ski resorts can sustain revenue by extending operating hours, improving access, and expanding winter amenities, but these measures may increase aggregate energy demand and deepen path-dependence risk when mitigation and transition planning remain weak. Resorts should therefore disclose both absolute emissions and intensity indicators, such as energy use per skier visit and emissions per lift-hour.

target-setting, resorts can select a recent pre-pandemic base year, such as 2019, when it reflects normal operations and aligns with Science Based Targets initiative (SBTi) guidance. A credible pathway would combine near-term emissions reduction, renewable electricity procurement, transport decarbonization, and transparent reporting through 2030, while aligning with Japan's 2050 net-zero policy framework (42–44).

#### Path transition and investment reallocation

Resorts near the Japan SRL-Deadline require particular attention because technical snowmaking and business-as-usual winter promotion provide limited durability under continued warming. Adaptation must therefore be embedded within a broader development pathway that combines mitigation, anticipation, and investment flexibility. In marginal pathways, resorts should prioritize convertible infrastructure during 2025–2030, shift investment toward low-carbon and non-snow recreation during 2030–2040, and progressively reduce dependence on skiing as the core economic anchor by 2040–2050. Certification or SDG-related initiatives should be connected to measurable changes in transport, energy, procurement, circularity, and local employment.

Anticipatory governance is especially important because maladaptation may also arise from continuity. Resorts can become locked into higher-risk pathways when they continue investing in snow-dependent facilities, parking-oriented access, winter-only branding, or high-throughput visitor models while snow reliability declines. The practical question is how each development pathway preserves future options, reduces emissions, and avoids shifting risk to workers, communities, ecosystems, or future investors.

### Theoretical contributions

This study contributes to understanding climate adaptation in Japan's ski tourism sector by connecting snow-reliability exposure, public resort actions, and maladaptation risk. First, it develops and applies a regionally calibrated SRL pathway framework that accounts for Japan's pronounced latitudinal and elevational gradients. The framework is grounded in JMA 1991–2020 station climatology and tested through multi-parameter sensitivity analysis, providing an empirical basis for adapting SRL screening beyond its European origins. The localized framework suggests that warming compresses the viable operating zone and shifts marginal sites toward snowmaking dependence or potential market withdrawal. The classification highlights the limits of technology-centered adaptation when it is not paired with structural transition.

Second, this study integrates the SRL pathway framework with Barnett and O'Neill's (7) maladaptation framework to construct a five-pathway typology (A1-B1-Dead, A1-B1-C1, A2-B1-C1, A2-B2-C1, A2-B2-C2) that links snow-reliability exposure to governance and investment risks. By cross-referencing pathway classifications with maladaptation codes (MA1, MA3, MA5), the study shows how similar actions, such as extended operating hours, access incentives, or infrastructure expansion, can carry different long-term implications depending on a resort's exposure and transition capacity. This pathway-contingent perspective aligns with recent scholarship that conceptualizes adaptation and maladaptation as a continuum shaped by timing, scale, governance context, mitigation effort, anticipation, and distributional outcomes (45).

Third, the study extends Barnett and O'Neill's (2010) framework by showing how continuity-driven exposure can be assessed alongside action-driven maladaptation. Where the original framework classifies observable actions with negative consequences, the present analysis suggests that maintaining snow-dependent business models without transition planning can also warrant maladaptation scrutiny when climatic exposure is high. This extension is relevant for climate adaptation governance because active maladaptation and delayed transition require different policy responses.

## Conclusions

This study develops an exploratory screening framework for linking snow-reliability exposure, resort development pathways, and maladaptation risk in Japan's ski tourism sector. First, the regionally calibrated SRL pathway framework captures Japan's pronounced latitudinal and elevational gradients. The JMA-grounded regional correction *Δ*z and multi-parameter sensitivity analysis provide a transparent empirical basis for adapting the SRL framework to Japan. The localized classification suggests that warming compresses the viable operating zone and shifts marginal sites toward snowmaking dependence or potential market withdrawal.

Second, analysis of 23 ski resorts affiliated with Protect Our Winters Japan demonstrates a polarized pathway structure. Approximately 61% of the sample falls into snowmaking-dependent or critically marginal categories, whereas only 13% occupies the most resilient category. This distribution suggests that climate change is reshaping the competitive landscape by concentrating long-term viability among geographically advantaged sites.

Third, integration of the maladaptation framework identifies systematic risks embedded in ostensibly adaptive measures. Increased greenhouse gas emissions risk concentrates in snowmaking-dependent resorts, where extended operating hours, enhanced accessibility, and reduced entry friction indicate higher aggregate energy-demand risk. Path dependence also emerges through fixed infrastructure, zoned terrain, rental systems, instruction systems, and winter-centered destination narratives.

These findings underscore the need for a governance shift from reactive save-the-season tactics toward proactive structural transition. The analysis suggests three linked levers: mitigation through year-round GHG accounting and transport decarbonization; adaptation through flexible, context-sensitive operating strategies; and anticipation through staged investment decisions that preserve future options. As warming continues and snow reliability declines, the choice is no longer whether to adapt, but how to adapt without deepening emissions, narrowing investment flexibility, or shifting burdens onto communities, ecosystems, and future generations.

### Limitations and future research directions

This study has several limitations. First, the SRL pathway classification is a literature-based and regionally calibrated screening framework. It uses elevation thresholds, a snowmaking buffer, and regional adjustments to compare exposure across resorts, but it does not replace direct modelling of snow depth, wet-bulb temperature, snowmaking windows, grooming, water availability, or resort-specific operating standards. Future research should combine this framework with multiannual meteorological observations and spatially explicit snowpack modelling. Second, the use of mean elevation as the representative resort altitude simplifies the vertical structure of ski areas. Sensitivity checks show that base and summit elevation assumptions can shift pathway classifications, so future work should use lift-power-weighted, slope-area-weighted, or spatially explicit elevation indicators where available. Third, the regional correction term *Δ*z should be validated against station-level and resort-level meteorological and snow observations. Fourth, the 23-resort POW Japan sample represents a small subset of a national sector that recent sources estimate at 481 ski areas in 2019 and 417 operating resorts in 2025 (39, 40), and it may over-represent sustainability-oriented operators. Fifth, maladaptation coding relied on public documents; interviews, financial data, energy-use records, snowmaking logs, and transport data would deepen insight into organizational decision-making. Finally, the framework treats resorts as analytical units even though ski areas may contain functionally interdependent upper and lower zones. Future research should combine resort-level policy analysis with spatial snowpack modelling, observed snow conditions, and comparative cases in other countries using Sustainable Resort Alliance or similar datasets. This source base may therefore under-detect distributive burdens (MA2) and the displacement or weakening of ongoing adaptation (MA4).

## Data Availability

The original contributions presented in the study are included in the article/Supplementary Material, further inquiries can be directed to the corresponding authors.
