# Supplementary material for: The climate change dilemma of ski destinations: adaptation becomes maladaptation
Source: Front Sports Act Living. 2026 Aug 18;8:1865169. doi: 10.3389/fspor.2026.1865169 (PMC13529999; doi:10.3389/fspor.2026.1865169)
Supplement: Supplementary file 1 [file Table1.docx]

Appendix A Ski Resort Lables and Official Names + Path and MA

| Resort Code | Resort_JP | Resort_EN | Path | MA1 | MA2 | MA3 | MA4 | MA5 |
| --- | --- | --- | --- | --- | --- | --- | --- | --- |
| JP01 | スキージャム勝山 | Ski Jam Katsuyama | A1-B1-Dead | 0 | 0 | 0 | 0 | 0 |
| JP02 | シーサイドバレースキー場 | Itoigawa Seaside Valley | A1-B1-Dead | 0 | 0 | 0 | 0 | 0 |
| JP03 | 舞子スノーリゾート | Maiko Snow Resort | A1-B1-Dead | 1 | 0 | 0 | 0 | 1 |
| JP04 | 湯沢中里スノーリゾート | Yuzawa Nakazato | A1-B1-Dead | 1 | 0 | 0 | 0 | 1 |
| JP05 | ムイカスノーリゾート | Muica Snow Resort | A1-B1-Dead | 0 | 0 | 0 | 0 | 0 |
| JP06 | キューピットバレイ | Cupid Valley | A1-B1-Dead | 1 | 0 | 0 | 0 | 1 |
| JP07 | ニノックススノーパーク | Ninox Snow Park | A1-B1-Dead | 1 | 0 | 0 | 0 | 1 |
| JP08 | エイブル白馬五竜 | Hakuba Goryu | A1-B1-C1 | 0 | 0 | 0 | 0 | 0 |
| JP09 | かたしな高原スキー場 | Katashina Kogen | A1-B1-C1 | 0 | 0 | 0 | 0 | 0 |
| JP10 | ニセコ東急グランドヒラフ | Niseko Tokyu Grand HIRAFU | A1-B1-C1 | 1 | 0 | 1 | 0 | 1 |
| JP11 | タングラム斑尾東急リゾート | Tangram Ski Circus | A1-B1-C1 | 0 | 0 | 1 | 0 | 0 |
| JP12 | 白馬岩岳スキー場 | Hakuba Iwatake | A1-B1-C1 | 0 | 0 | 0 | 0 | 0 |
| JP13 | 斑尾高原スキー場 | Madarao Kogen | A1-B1-C1 | 0 | 0 | 0 | 0 | 0 |
| JP14 | 野沢温泉スキー場 | Nozawa Onsen | A1-B1-C1 | 1 | 0 | 0 | 0 | 1 |
| JP15 | 白馬八方尾根スキー場 | Hakuba Happo-one | A2-B1-C1 | 0 | 0 | 0 | 0 | 0 |
| JP16 | ハンターマウンテン塩原 | Hunter Mountain Shiobara | A2-B1-C1 | 0 | 0 | 0 | 0 | 0 |
| JP17 | たんばらスキーパーク | Tambara Ski Park | A2-B1-C1 | 0 | 0 | 0 | 0 | 0 |
| JP18 | 東急リゾートタウン蓼科 | Tateshina Tokyu | A2-B1-C1 | 0 | 0 | 0 | 0 | 0 |
| JP19 | 岩手網張温泉スキー場 | Amihari Onsen | A2-B1-C1 | 0 | 0 | 0 | 0 | 0 |
| JP20 | 木曽福島スキー場 | Kiso Fukushima | A2-B2-C1 | 0 | 0 | 0 | 0 | 0 |
| JP21 | 白樺高原国際スキー場 | Shirakaba Kogen Kokusai | A2-B2-C2 | 0 | 0 | 0 | 0 | 0 |
| JP22 | しらかば2in1 | Shirakaba 2in1 | A2-B2-C2 | 0 | 0 | 0 | 0 | 0 |
| JP23 | 御嶽スキー場 | Ontake Ski Area | A2-B2-C2 | 0 | 0 | 0 | 0 | 0 |

Note: Internal identifiers (JP01–JP23) are used for indexing and cross-referencing only; they carry no implications of ranking or weighting. MA columns (MA1–MA5) indicate the presence (1) or absence (0) of maladaptation risks based on policy text coding; each resort is counted at most once per MA category, regardless of the number of policy actions identified within that category.
